# Transcutaneous Electrical Nerve Stimulation vs Transcutaneous Electrical Nerve Stimulation With Music in Persistent Low-Back Pain: Protocol for a Pilot Feasibility Trial

**DOI:** 10.2196/82382

**Published:** 2026-02-24

**Authors:** Seema Radhakrishnan, Michael Glynn, Anne-Marie Robertson, Anna Fedyczkowski, Lieu Trinh, Sabrina Naz

**Affiliations:** 1 Pain Management Centre, Westmead Hospital, Cnr Darcy Road and Hawkesbury Road, Westmead, 2145, Australia, 61 288904597, 61 288904598; 2 Westmead Clinical School, University of Sydney, Sydney, Australia; 3 School of Population Health, University of New South Wales, Sydney, Australia; 4 Department of Geriatrics and Rehabilitation, Fairfield Hospital, Prairiewood, Australia; 5 Pain Management Centre, Western Sydney Local Health District, Research and Education Network (REN), Westmead, Australia

**Keywords:** transcutaneous electrical nerve stimulation, TENS, music therapy, chronic pain, pilot trial, spinal pain

## Abstract

**Background:**

Transcutaneous electrical nerve stimulation (TENS) works on the principle of the gate control theory of pain and is used as a nonpharmacological pain management intervention. Music therapy or listening to self-selected music has also been shown to reduce pain intensity, anxiety, and depression. There has been no published literature that has explored whether these modalities can work synergistically.

**Objective:**

This study aims to test the feasibility of a clinical trial comparing TENS alone to TENS combined with self-selected music in participants with persistent low-back pain lasting more than 3 months.

**Methods:**

This will be a prospective, randomized controlled pilot study to compare TENS alone vs TENS combined with participant self-selected music. We hope to enroll 20 participants. The participants will be their own controls, and the order in which the intervention is delivered will be determined by randomization.

**Results:**

The study is open for recruitment from August 2025. The results are expected in 18 months from the start of recruitment. The primary outcome will be the feasibility of the intervention, monitored by adherence rate, dropout rate, and the percentage of the eligible population that consented. Secondary outcomes collected will include pain intensity; mood using the 21-item Depression, Anxiety, and Stress Scale; medication consumption; participant satisfaction; and adverse reactions.

**Conclusions:**

If the pilot trial is feasible and indicates a positive synergistic trend with TENS and music, the trial will help to inform the feasibility for a larger trial with more varied pain diagnoses, including acute and chronic pain.

## Introduction

### Background

Low-back pain is the leading cause of disability across the globe [1]. Pharmacological interventions such as opioids, when used as a long-term management strategy for persistent spinal pain, can result in significant adverse effects [2]. Nonpharmacological noninvasive interventions that facilitate endogenous pain modulatory pathways have the potential to provide improvement in pain and function while minimizing adverse events [3].

Transcutaneous electrical nerve stimulation (TENS) is a nonpharmacological method to manage both acute and persistent pain that works on the principle of gate theory. TENS uses electrical stimulation delivered through surface electrodes encased in a patch to activate large afferent fibers, which causes presynaptic inhibition of afferent nociceptive input at the dorsal horn of the spinal cord [3]. TENS also activates central inhibitory pathways in the brain and spinal cord and acts through multiple neurotransmitters involving opioid, gamma-aminobutyric acid, serotonin, muscarinic, and cannabinoid receptors [4]. Analgesia following TENS can last beyond the period of application for up to 24 hours [5]. There is some evidence that the type of TENS used can impact the duration of analgesia, with higher frequency, lower intensity, or conventional TENS providing faster analgesia but less poststimulation analgesia and low frequency, high intensity, or acupuncture such as TENS providing longer poststimulation analgesia, which can last several hours after the stimulation is terminated [6]. Burst mode TENS, which delivers pulses of electrical stimulation in bursts, is thought to combine the effects of the abovementioned 2 modes and provide quicker onset analgesia that lasts longer than the duration of the stimulation [7]. TENS has been shown to improve functional disability in persistent low-back pain [8].

Music listening interventions, such as music therapy delivered by trained music therapists or listening to self-selected music, have been shown to reduce pain intensity, anxiety, and depression [9]. Functional neuroimaging indicates that this could be due to increasing the descending inhibition as well as changes to the salient stimuli detection circuitry involving somatosensory and prefrontal areas, amygdala, and cingulate cortex, as well as the insula. There is also evidence that reward pathways involving dopamine play a part in the analgesic and mood-altering effects of music listening interventions [10]. Cognitive mechanisms involved in analgesia and mood-altering effects of music listening interventions may include sustained attention on the music rather than pain, associations of self-selected music to pleasant memories, and agency related to selection of the type of music and the volume at which it is played [9]. Listening to self-selected music as well as engaging with the content of the music, or active listening, may reduce pain intensity and improve emotional distress more than music therapy [11]. Listening to self-selected music could have the potential advantage of improved self-efficacy and motivation, increased engagement in daily activities, and incorporation as a cost-effective noninvasive component to the overall pain management strategy [9].

There are no studies in the published literature that combine the abovementioned 2 nonpharmacological interventions in pain management. There is potential for the 2 interventions to act synergistically, providing superior pain relief and improved function compared with either intervention alone, through engagement of central pain modulation pathways and activation of descending inhibitory mechanisms.

### Aims

The primary aim of this study is to test the feasibility of a clinical trial comparing TENS alone to TENS combined with listening to self-selected music in participants with low-back pain with or without leg pain lasting more than 3 months. The results of this feasibility study will be used to inform a larger trial of these interventions in participants with persistent neck and low-back pain lasting more than 3 months. A larger study will be considered feasible if 80% or more of the eligible population consents to the study and 80% or more of the participants are able to adhere to the protocol. The secondary aims are to explore if combining the 2 modalities has a synergistic effect on analgesia and function. A difference of 2 points in the numerical rating scale for pain intensity will be considered significant.

### Hypotheses

First, we hypothesize that administration of TENS combined with music will be equally feasible compared to TENS alone and will be well tolerated by participants. Second, we hypothesize that TENS combined with listening to self-selected music will have improved analgesia compared to using TENS alone.

## Methods

### Overview

**Figure 1.**
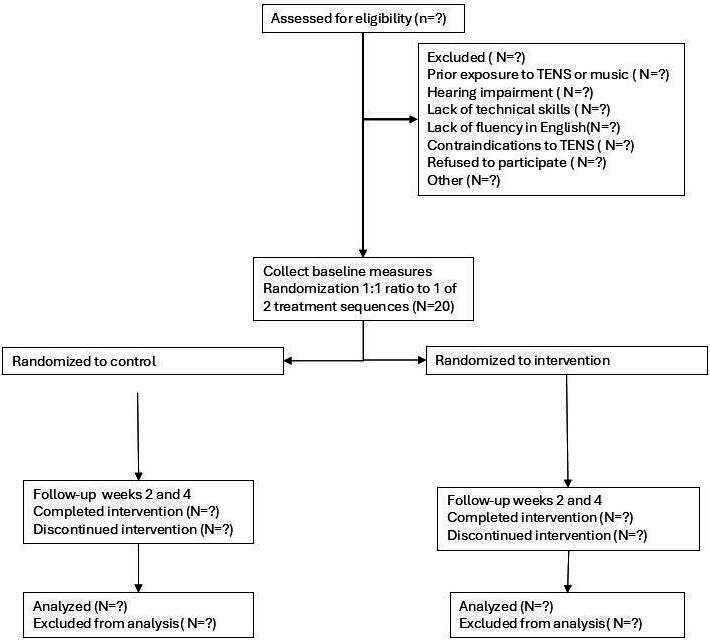
CONSORT (Consolidated Standards of Reporting Trials) diagram: TENS with music pilot study. TENS: transcutaneous electrical nerve stimulation.

TENS with music study will be a prospective, randomized controlled pilot study to compare TENS alone to TENS combined with participant self-selected music. The participants will be randomized to receive 2 weeks of TENS therapy and 2 weeks of TENS with self-selected music using a streaming service, CD player, Long Play, or radio. The order in which this is delivered will be determined by randomization. Therefore, 1 group will receive standard TENS for 2 weeks, followed by TENS with self-selected music for 2 weeks. This order will be reversed in the other group with no washout period. The participants will be compared against themselves for the outcomes, thus reducing the number of participants to power the study. We are hoping to recruit 20 participants for this pilot trial (Figure 1). We will use TENS and TENS with music as part of a holistic pain management plan involving pain neuroscience education, behavior activation, and coping skills training that is individually tailored and not as a stand-alone intervention for pragmatic reasons.

### Eligibility

All participants aged 18 years or older, presenting with low-back pain lasting more than 3 months with or without referred or radicular limb pain, and attending a pain clinic at a metropolitan tertiary hospital in Australia will be eligible to participate in the study. Participants who have used TENS or music therapy in the past to manage their pain and those participants with a significant hearing impairment, the lack of technical ability to play music through a streaming platform or operate other music listening devices such as an LP or CD player, or an inability to communicate in English will be excluded from participation. Individuals with contraindications to the use of a TENS machine, such as having a permanent cardiac pacemaker, implantable cardioverter defibrillator, or neurostimulators, will also be excluded [12].

### Treatment

TENS will be administered for 1 hour at a time, up to a maximum of 6 times every 24 hours. Participants are free to select from available modes, which include burst, continuous, or modulate. Other parameters, such as pulse width, pulse rate, and pulse intensity, can also be modified by the participant. The pulse intensity of the TENS machine can be adjusted from 0 to 80 mA, and the pulse frequency from 2 to 150 Hz. The aim is to use the frequencies, amplitude, and mode to provide a comfortable paresthesia [13]. The participants can vary the electrode placement to provide the best possible analgesia.

The TENS will be self-administered. Music from a self-selected playlist from a set of genres such as classical, easy listening, pop, rhythm and blues, vocal, and musical will be used. We will use a participant diary to record the duration of TENS each day and the type of music listened to. Music can be listened to with or without headphones. Participants will be provided with a 1-month Spotify (Spotify AB) subscription voucher to enable ad-free music listening.

### Randomization

Participants will be randomized in a 1:1 ratio to 1 of 2 treatment sequences:

- Sequence AB: 2 weeks of standard TENS followed by 2 weeks of TENS combined with self-selected music
- Sequence BA: 2 weeks of TENS combined with self-selected music followed by 2 weeks of standard TENS

Randomization will be performed using a computer-generated allocation list created in Stata (StataCorp LLC) before study commencement. A permuted block design with variable block sizes will be used to ensure balance between sequences throughout recruitment. The randomization seed and code will be archived to allow reproducibility. Allocation concealment will be maintained by storing the randomization list in the Research Electronic Data Capture (REDCap; Vanderbilt University) randomization module, which will reveal the assigned sequence only after a participant has provided informed consent and completed baseline assessments.

The randomization result will be communicated to participants by telephone within 24 hours of their initial assessment. Investigators responsible for outcome collection and statistical analysis will remain blinded to treatment sequence. Participants cannot be blinded due to the pragmatic nature of the intervention.

### Outcomes

The primary outcome will be the feasibility of the intervention, measured as adherence rate, dropout rate, and the percentage of the eligible population that consented. Adherence rates will include the number of participants using TENS alone or TENS with music regularly more than once per week, adherence to filling out the pain diaries and other patient-reported outcome measures, and attendance at follow-up clinic visits.

Secondary outcomes include the following:

- Pain intensity as a numerical rating scale (NRS) score collected before and after each session using a pain diary. The baseline pain will be collected for 1 week before the intervention when they initially attend the clinic.
- Opioid and other analgesic consumption over a 24-hour period collected in the pain diary
- Participant satisfaction using a 5-point Likert scale
- Mood measured through the 21-item Depression, Anxiety, and Stress Scale
- Function measured through the Patient-Specific Functional Scale [14]
- Compliance with other interventions, such as participation in interdisciplinary pain programs
- Adverse outcomes, including worsening pain and skin reaction to the electrodes used in TENS

**Figure 2.**
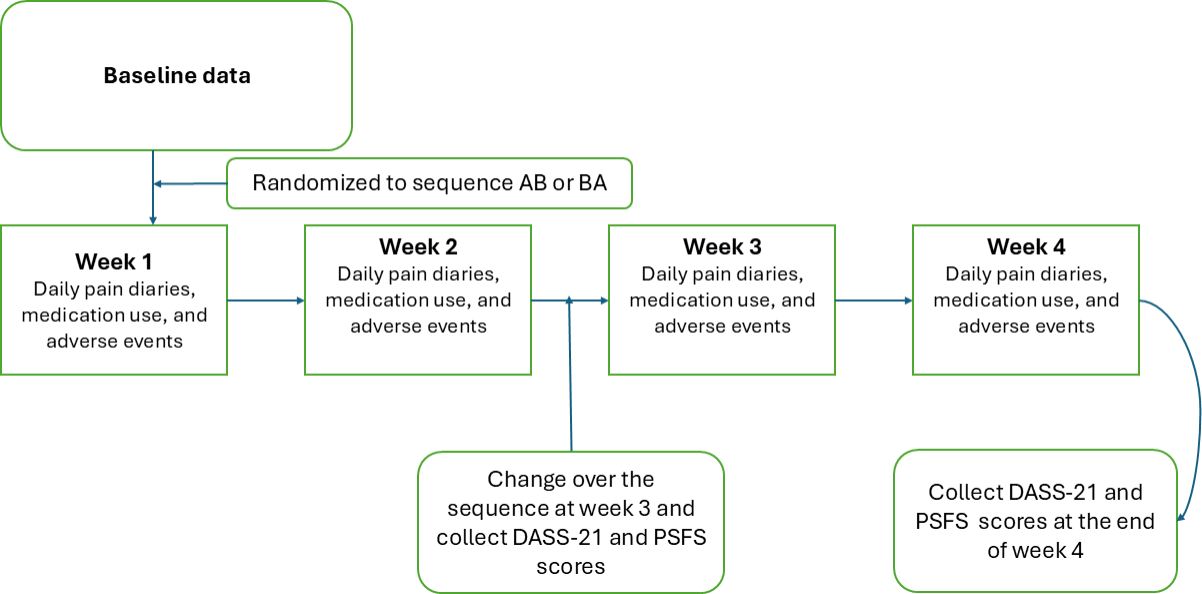
Workflow for transcutaneous electrical nerve stimulation with music pilot study. DASS-21: 21-item Depression, Anxiety, and Stress Scale; PSFS: Patient-Specific Functional Scale.

All reported adverse reactions will also be collected through the study period when using the TENS machines through entries in the pain diary as well as during interviews with the participants. The self-reported outcome measures, such as the 21-item Depression, Anxiety, and Stress Scale and the Patient-Specific Functional Scale, will be recorded through REDCap forms during the clinic appointments. Figure 2 summarizes the process.

### Blinding

The observer who collates the data and the statistician who analyzes the results will be blinded. The participants will not be blinded, as blinding is not pragmatic for this intervention. The equipment required includes a TENS machine, a pain diary, and the participants’ own device for listening to music on Spotify, CD, LP, radio, or other music listening devices.

### Data Collection

A standardized pain diary (Multimedia Appendix 1) will be used for collecting the primary outcome and some of the secondary outcomes, such as medication taken over 24 hours. Specific forms will be used for other secondary outcomes, which are available through REDCap. The data will be stored in a password-protected, secure database via REDCap. The data collected will be deidentified and assigned a study code. The pain management unit will be the custodian of the data stored in the password-protected drive within the local health district computer, with access restricted to the investigators only. The data will be stored for 7 years after the completion of the study. Table 1 summarizes the data collection points.

**Table 1.** Data collection points.

Measure	Frequency	Collection points
Pain diaries	Daily	Baseline, week 1, week 2, week 3, and week 4
Average pain over 24 hours (numerical rating scale)	Weekly	Baseline, week 1, week 2, week 3, and week 4
Medications	Weekly	Baseline, week 1, week 2, week 3, and week 4
Patient-Specific Functional Scale	Fortnightly	Baseline, week 2, and week 4
21-item Depression, Anxiety, and Stress Scale	Fortnightly	Baseline, week 2, and week 4
Pain sites update	1 time	Baseline
Pain duration	1 time	Baseline
Demographics	1 time	Baseline

### Power Calculation

Power was planned for the primary within-subject comparison (paired difference between TENS with self-selected music and TENS), with a 2-sided *α*=.05 and 80% power. In the absence of previous variance data, sensitivity analyses were conducted across standardized paired effect sizes (dz). The required numbers of completed pairs (participants who complete both study periods) are presented in Table 2.

**Table 2.** Sample size calculation.

Effect size (dz)	Completed pairs needed, n	Recruits with 10% dropout, n	Recruits with 20% dropouts, n	Recruits with 30% dropouts, n
0.4	52	57	62	68
0.5	34	37	41	44
0.6	24	26	29	31
0.7	19	21	23	25
0.8	15	17	18	20

As a pilot study (target recruitment N=20), the trial is adequately powered only for larger effects (approximately dz≥0.70). Therefore, the primary purpose of this pilot study is to assess the feasibility, estimate variance, and provide effect size estimates to inform the definitive sample size for the main trial.

All calculations were performed using G*Power version 3.1 (Heinrich Heine University), which is widely used for sample size and power analysis in clinical research.

### Statistical Methods

This study is a 2-period, 2-sequence crossover pilot trial without a washout period between treatments. Analyses will primarily focus on feasibility outcomes (recruitment, adherence, data completeness, and dropout) using descriptive statistics (means, medians, and proportions) with 95% CIs rather than formal hypothesis testing. These feasibility metrics will inform the practicality of conducting a larger definitive trial.

For the primary clinical outcome of pain intensity, exploratory analyses will first be conducted using paired *t* tests to compare mean scores between TENS and TENS with self-selected music within participants. Normality of paired differences will be assessed using graphical methods and the Shapiro-Wilk test. If assumptions are violated, the Wilcoxon signed-rank test will be applied as a nonparametric alternative. Categorical variables (eg, adverse events and compliance categories) will be analyzed initially using chi-square tests, or Fisher exact test if expected cell counts are small. All tests will be 2-sided with a significance level of *α*=.05.

Following these preliminary analyses, formal modeling tailored to the crossover design will be undertaken. Linear mixed-effects models will be specified with fixed effects for treatment (TENS vs TENS with self-selected music), period, and sequence, and a random intercept for participant to account for within-participant correlations. Because no washout period is present, carryover effects will be explicitly assessed by including a prior-treatment term and by examining period×sequence interactions. If evidence of carryover is observed, analyses will be restricted to first-period data as a sensitivity check, treating it as a parallel-group comparison.

Secondary outcomes (mood, function, satisfaction, medication use, and adverse events) will be analyzed using appropriate mixed-effects regression models: linear for continuous outcomes, cumulative logit for ordinal outcomes, negative binomial for counts, and logistic for binary outcomes. These analyses are exploratory in nature. Given the pilot design and small sample size, no formal correction for multiple testing will be applied; instead, effect sizes and CIs will be reported to guide future sample size calculations.

Intention-to-treat principles will be followed, analyzing participants according to their randomized sequence regardless of adherence or protocol deviations. Mixed-effects models allow inclusion of all available data, including partial data from participants who drop out after the first period, thereby minimizing bias. Per-protocol analyses will be conducted as sensitivity checks. Missing data will be handled descriptively, with analyses restricted to available cases. No multiple imputation will be attempted due to the small sample size.

As a feasibility study with 20 participants, the trial is powered only to detect large effects (approximately dz≥0.70). The observed variance and effect sizes will be used to refine sample size calculations for the definitive trial.

All analyses will be conducted using Stata and SPSS Statistics (IBM Corp).

### Ethical Considerations

The trial has been approved by the local health district ethics committee (2024/PID02885 - 2024/ETH02492) and is currently screening participants for recruitment. Written informed consent will be obtained from all participants. The participants will be informed that a withdrawal of consent is possible at any point and that their clinical care will not be compromised. The participants will not receive any remuneration.

## Results

This manuscript describes the protocol for an ongoing pilot trial, with results to be reported upon trial completion. Recruitment for the trial began in August 2025. As of manuscript submission, 4 participants have been enrolled, with a target of 20 participants by the end of August 2026. Data analysis is expected to be completed by December 2026. The results are planned to be published as an open-access article in 2027 and used to inform the design of a larger trial.

A summary of the results will be disseminated to the participants.

## Discussion

### Anticipated Findings

This pilot study primarily aims to assess the feasibility of combining TENS with self-selected music. The anticipated results include approximately 80% adherence to daily use of TENS or TENS combined with self-selected music and completion of daily diaries on 80% of the study days. An adherence rate of 80% will be considered sufficient for the current data collection methods to be deemed feasible for a larger trial. It is hoped that adherence to data collection procedures, including real-time documentation of pain scores and medications used, will be evaluated among participants [15]. The trial is not likely to reveal any additive analgesic benefit for TENS and self-selected music due to the small participant numbers, but it is anticipated that a trend toward improved pain scores, better function, and reduced analgesia requirements by combining the 2 modalities may be revealed. A change of 2 points in the 11-point scale on the NRS will be most clinically relevant, but even a point difference in the NRS can be considered as the minimal clinically significant change to the participants [16]. A change in pain intensity of 2 points in the NRS is accepted as best correlated with general patient satisfaction and other meaningful outcome measures in chronic low-back pain [17]. The clinically meaningful change will differ by baseline pain; however, for pragmatic reasons in this feasibility trial, it was decided to not stratify participants based on baseline pain and adopt the general threshold. It is hoped that the acceptability of combining TENS with self-selected music as an intervention for pain management will be determined, and whether the outcome measurements and methods are perceived as burdensome and time-consuming will be assessed in this small sample. Additional insights are expected regarding noncompliance with the treatment, which may include lack of perceived improvement in pain or adverse events associated with use, such as worsening pain or skin irritation from the electrodes, as well as other factors such as lack of motivation [18].

Chronic pain is a complex condition, and the intensity of pain and the functional limitations experienced by people with chronic pain are best managed using a biopsychosocial framework [19]. The use of TENS and self-selected music will fit within this framework, along with education and an interdisciplinary pain self-management program, to achieve person-centered functional goals by addressing the multiple contributors to a person’s pain experience [20]. The 2-week time frame for the intervention, rather than an in-clinic comparison, will provide a more pragmatic approach to test the real-world efficacy of combining the interventions [21]. Focusing not only on improvement of pain scores but also on other aspects of pain management, including mood, function, and medication use, will provide a comprehensive picture of the interaction between the 2 modalities.

The self-selection of the pulse amplitude, frequency, and mode is recommended due to the complexity of the pain experience. TENS provides optimal analgesia when the delivered sensation is comfortable for the person experiencing pain [22]. Self-selection of the TENS parameters and music also has the potential to increase the sense of control of the participants, thus improving self-efficacy, which can also reduce pain intensity and improve compliance [23]. Due to constraints related to resources and time, a smaller number of participants were selected, as this is a feasibility trial. A crossover trial design was chosen where participants were their own controls to reduce the sample size [24]. We chose not to have a washout period due to the short-acting nature of the intervention, with evidence that postuse analgesia usually wears off by 24 hours for TENS [22].

The duration of analgesia from music listening interventions in chronic pain is unclear, but available evidence indicates that it is short-lived and does not last hours or days beyond the session [25]. The clinical trials using TENS do not consistently report adherence rates and can vary from 50% to 80% [18]. We chose the higher adherence rate, as this is a feasibility study. Those with previous exposure to TENS or music listening interventions were excluded due to the potential for placebo effects arising from previous experience [26]. Stratification of participants based on their previous use of TENS or music listening interventions could have reduced this bias in recruitment. However, given the small sample size and the pilot feasibility nature of this study, stratification would have introduced unnecessary complexity and was not aligned with the primary objective, which was to assess the feasibility of data collection methods [27].

### Limitations

The study requires the participants to fill out a significant amount of information daily, which may contribute to participant burden. It also increases the risk of noncompliance with regular entries and introduces recall bias if participants try to record the information before review. One of the main limitations of the study will be the lack of concurrent electronic monitoring to complement the participant-reported data. The study methodology also does not allow for blinding of the participants due to the crossover design. We have excluded participants who cannot communicate in English, those who cannot use the TENS machine due to functional limitations, and those who are unable to use streaming technology or other technology that plays music for pragmatic reasons in this feasibility trial. The research team acknowledges that this will affect the generalizability of the results by excluding culturally diverse cohorts and those who are less tech savvy. However, such individuals are expected to represent only a small proportion of otherwise eligible participants. This point will be addressed if a larger trial is considered feasible. Participants have considerable flexibility in choosing the frequency, mode, and electrode placement when using TENS, as well as the type of music they listen to. While this variability makes it challenging to determine which parameters were truly effective, predict underlying mechanisms, and replicate results, these limitations are acknowledged. As this study primarily focuses on feasibility, such factors will be carefully addressed when designing a larger trial. The pilot study is designed to detect only large effect sizes and to inform power calculations for future trials, and it may fail to detect small effect sizes. It is anticipated that approximately 90% of participants will need to demonstrate a positive trend, defined as at least a 1-point improvement on the NRS, with the combination of TENS and self-selected music, to calculate the participant numbers for a larger trial that is adequately powered to detect a moderate effect size [28]. However, the authors anticipate that the effect sizes and the CIs from the pilot study will help to calculate a conservative estimate to detect smaller effect sizes from a larger definitive trial [29].

### Future Directions

If the trial is feasible and indicates a positive synergistic trend with TENS and self-selected music, it will help to plan for a larger trial with more varied pain diagnoses, including acute and chronic pain other than spinal pain. The aim of a larger study will be to show if combining the 2 modalities of music and TENS will improve pain intensity and function more than TENS alone. The pilot will test the feasibility of using pain diaries as a reliable indicator of gathering information in a larger trial. Future directions could include automated methods for data collection, which could provide more reliable indicators of adherence, function, and medication use.

## Supplementary material

10.2196/82382 Multimedia Appendix 1 Data collection template.

## Abbreviations

NRS: numerical rating scale
REDCap: Research Electronic Data Capture
TENS: transcutaneous electrical nerve stimulation
