# Supplementary material for: Transcutaneous Electrical Nerve Stimulation vs Transcutaneous Electrical Nerve Stimulation With Music in Persistent Low-Back Pain: Protocol for a Pilot Feasibility Trial
Source: JMIR Res Protoc. 2026 Feb 24;15:e82382. doi: 10.2196/82382 (PMC12931921; doi:10.2196/82382)
Supplement: Multimedia Appendix 1 [file resprot-v15-e82382-s001.docx]

**Pain diary (C)**

**Completing the pain diary will help the study team to see if the intervention was helpful to reduce the pain levels.**

**Instructions**

1) At various times during the day, as often as possible, mark the line at the spot that reflects your pain levels at that time. The scale is from 0 to 10, with 0 representing “no pain” and 10 representing the “worst pain” possible. Please record if you used TENS , and for how long each time

2) Record the activity that you were doing at the time (e.g. sitting, doing housework, sleeping, shopping, gardening etc).

3) Record any pain medicine taken, at the time that you took it.

4) At the bottom of the page record any adverse events/ general comments about your sleep/ feelings for the day

5) Also don’t get up and record during the night- only do so if you are awake or complete from memory the next morning

**Day & Date**

**Pain score: 0 to 10, 0 is no pain and 10 is the worst pain**

**TENS machine mode used: Burst/Continuous/Modulate**

| **Time** | **Pain score** | **Used TENS**  **(Yes/No)**  **If yes please indicate duration it was used** | **What were you doing when using the TENS**  **(Resting/other activities)** | **Other coping**  **Techniques used** | **Medicine taken**  **Please write the name and dose** |
| --- | --- | --- | --- | --- | --- |
| **6am** |  |  |  |  |  |
| **7am** |  |  |  |  |  |
| **8am** |  |  |  |  |  |
| **9am** |  |  |  |  |  |
| **10 am** |  |  |  |  |  |
| **11am** |  |  |  |  |  |
| **12 pm** |  |  |  |  |  |
| **1 pm** |  |  |  |  |  |
| **2pm** |  |  |  |  |  |
| **3 pm** |  |  |  |  |  |
| **4 pm** |  |  |  |  |  |
| **5 pm** |  |  |  |  |  |
| **6 pm** |  |  |  |  |  |
| **7 pm** |  |  |  |  |  |
| **8 pm** |  |  |  |  |  |
| **9 pm** |  |  |  |  |  |
| **10 pm** |  |  |  |  |  |
| **11 pm** |  |  |  |  |  |
| **12 am** |  |  |  |  |  |
| **1 am** |  |  |  |  |  |
| **2 am** |  |  |  |  |  |
| **3 am** |  |  |  |  |  |
| **4 am** |  |  |  |  |  |
| **5am** |  |  |  |  |  |
|  |  |  |  |  |  |

**Please note if you had any adverse effects from the TENS treatment today:**

**Any other comments**

**Pain diary(I)**

**Completing the pain diary will help the study team to see if the intervention was helpful to reduce the pain levels.**

**Instructions**

1) At various times during the day, as often as possible, mark the line at the spot that reflects your pain levels at that time. The scale is from 0 to 10, with 0 representing “no pain” and 10 representing the “worst pain” possible. Please record if you used TENS, and for how long each time

2) Record the activity that you were doing at the time (e.g. sitting, doing housework, sleeping, shopping, gardening etc).

3) Record any pain medicine taken, at the time that you took it.

4) At the bottom of the page record any adverse events/ general comments about your sleep/ feelings for the day

5) Also don’t get up and record during the night- only do so if you are awake or from memory the next morning

6) Please record the type of music you listed (list below) to and for how long

**Music genres:**

**Day & Date**

**Pain score: 0 to 10, 0 is no pain and 10 is the worst pain.**

**TENS machine mode used: Burst/Continuous/Modulate**

| **Time** | **Pain score** | **Used TENS**  **(Yes/No)**  **If yes please indicate duration it was used** | **Did you use music with TENS. If yes please select the genre (more than one)** | **What were you doing when using the TENS**  **(Resting/other activities)** | **Other coping**  **Techniques used** | **Medicine taken**  **Please write the name and dose** |
| --- | --- | --- | --- | --- | --- | --- |
| **6am** |  |  |  |  |  |  |
| **7am** |  |  |  |  |  |  |
| **8am** |  |  |  |  |  |  |
| **9am** |  |  |  |  |  |  |
| **10 am** |  |  |  |  |  |  |
| **11am** |  |  |  |  |  |  |
| **12 pm** |  |  |  |  |  |  |
| **1 pm** |  |  |  |  |  |  |
| **2pm** |  |  |  |  |  |  |
| **3 pm** |  |  |  |  |  |  |
| **4 pm** |  |  |  |  |  |  |
| **5 pm** |  |  |  |  |  |  |
| **6 pm** |  |  |  |  |  |  |
| **7 pm** |  |  |  |  |  |  |
| **8 pm** |  |  |  |  |  |  |
| **9 pm** |  |  |  |  |  |  |
| **10 pm** |  |  |  |  |  |  |
| **11 pm** |  |  |  |  |  |  |
| **12 am** |  |  |  |  |  |  |
| **1 am** |  |  |  |  |  |  |
| **2 am** |  |  |  |  |  |  |
| **3 am** |  |  |  |  |  |  |
| **4 am** |  |  |  |  |  |  |
| **5am** |  |  |  |  |  |  |
|  |  |  |  |  |  |  |

**Please note if you had any adverse effects from the TENS treatment today:**

| **NO** | **Music Genres** | **examples** |
| --- | --- | --- |
| 1. | Classical | Paco belle cannons |
| 2. | Easy listening | Any individual and or band you like to hear |
| 3. | POP | 70s, 80s, 90s hits |
| 4. | Rhythm & Blues | Steve wonder |
| 5. | Vocal | Any individual you like to hear sing |
| 6. | Musical | Cats, Phantom of the opera, Les Misérables |

**Any other comments**
